# Fruit removal increases root-zone respiration in cucumber

**DOI:** 10.1093/aob/mcu192

**Published:** 2014-10-09

**Authors:** H.-P. Kläring, I. Hauschild, A. Heißner

**Affiliations:** Leibniz Institute of Vegetable and Ornamental Crops, Theodor-Echtermeyer-Weg 1, D-14979 Groβbeeren, Germany

**Keywords:** Cucumber, *Cucumis sativus*, diffuse exudation, fruit load, microbial activity, photosynthesis, root zone CO_2_ gas exchange, sink–source relations, soluble carbohydrates, transpiration

## Abstract

**Background and Aims:**

Many attempts have been made to avoid the commonly observed fluctuations in fruit initiation and fruit growth in crop plants, particularly in cucumber (*Cucumis sativus*). Weak sinks of the fruit have been assumed to result in low sink/source ratios for carbohydrates, which may inhibit photosynthesis. This study focuses on the effects of low sink–source ratios on photosynthesis and respiration, and in particular root-zone respiration.

**Methods:**

Mature fruit-bearing cucumber plants were grown in an aerated nutrient solution. The root containers were designed as open chambers to allow measurement of CO_2_ gas exchange in the root zone. A similar arrangement in a gas-exchange cuvette enabled simultaneous measurements of CO_2_ exchange in the shoot and root zones.

**Key Results:**

Reducing the sinks for carbohydrates by removing all fruit from the plants always resulted in a doubling of CO_2_ exchange in the root zone within a few hours. However, respiration of the shoot remained unaffected and photosynthesis was only marginally reduced, if at all.

**Conclusions:**

The results suggest that the increased level of CO_2_ gas exchange in the root zone after removing the carbon sinks in the shoot is due primarily to the exudation of organic compounds by the roots and their decomposition by micro-organisms. This hypothesis must be tested in further experiments, but if proved correct it would make sense to include carbon leakage by root exudation in cucumber production models. In contrast, inhibition of photosynthesis was measurable only at zero fruit load, a situation that does not occur in cucumber production systems, and models that estimate production can therefore ignore (end-product) inhibition of photosynthesis.

## INTRODUCTION

Many endeavours have been made to avoid the commonly observed fluctuations in fruit initiation and fruit growth, particularly in cucumber (*Cucumis sativus*), where the fruit is the main sink organ for carbohydrates (Marcelis, 1992). For this reason, weak sinks of the fruit were assumed to result in low sink/source ratios for carbohydrates, which may inhibit photosynthesis. This feedback inhibition has often been observed in small plants under laboratory conditions, particularly in typical starch storers such as cucumbers (Goldschmidt and Huber, 1992). With intensively grown adult cucumber plants, however, Marcelis (1991) observed no reduction in leaf photosynthesis until 16 d after the fruit was removed, whereas thereafter photosynthesis and dry matter growth fell sharply. He also found no increase in the concentrations of sugar and starch in the leaves (Marcelis, 1991).

Wasting carbohydrates through increased respiration, particularly in the alternative respiratory pathway (Lambers, 1982), could be one of the ways in which plants avoid the early (end-product) inhibition of photosynthesis. A number of reports examine this possibility (Millenaar and Lambers, 2003). Another possible process, namely exudation as a carbohydrate overflow, has yet to be considered. It has been reported that increasing the source/sink ratio by raising the CO_2_ concentration in the air increases root exudation (Hill *et al*., 2007; Phillips *et al*., 2009). However, to our knowledge there have been no investigations of the effect of limited sinks in the shoot on root respiration and exudation. Mechanistic models, even highly sophisticated ones, that describe the growth of commercial greenhouse crops in order to improve climate control and plant management include neither a possible feedback mechanism of sink limitation on photosynthesis nor a loss of carbohydrates by root exudation (e.g. Marcelis *et al*., 1998; Trouwborst *et al*., 2011).

In order to address this problem, the effect of sink–source relations on CO_2_ release in the root zone of hydroponically grown cucumber, namely release originating from root respiration and the microbial decomposition of organic compounds released by the roots, was investigated.

## MATERIALS AND METHODS

### Cultivation of plants

Two experiments were conducted in a greenhouse measuring root-zone respiration (RZR1, RZR2) in addition to four in a CO_2_ gas-exchange cuvette (C1, C2, C3, C4). Cucumber plants (*Cucumis sativus* ‘Torreon’; Enza Zaden, Enkhuizen, The Netherlands) were used in all experiments. The seeds were germinated in gravel and pricked out into pots containing gravel. Once the third true leaf had unfolded, the gravel was rinsed from the roots. The plants were then hung by their shoots on a wire, and the roots were set in polyethylene containers with an aerated nutrient solution. The nutrient solution was prepared according to the recommendations for hydroponic production (De Kreij *et al*., 1997). The plants then continued to grow in a greenhouse. The temperature set points for heating were 20 °C during the day and 18 °C at night; the greenhouse was ventilated when the temperature reached 27 °C or when the relative humidity exceeded 80 %.

For experiments in the CO_2_ gas-exchange cuvette, apical tissue beyond the 12th leaf was removed from all plants. The lowermost leaf was removed when the plants were transferred to the cuvette. The plants in the greenhouse experiments were terminated at a height of 2 m, leaving 20 (experiment RZR1) or 18 (experiment RZR2) leaves. All axillary shoots, with the exception of the uppermost two, were removed as soon as they appeared. The fruit set in the first five axils was removed; one fruit was allowed to grow in each subsequent axil.

### Measurement of CO_2_ gas exchange in the root zone in greenhouse experiments

A system for measuring CO_2_ gas exchange in adult plants in a greenhouse was designed following the general principle of open chambers. It consisted of 24 polyethylene containers, an air supply system and an infrared CO_2_ gas analyser (LI 820; LI-COR, Lincoln, USA). Each 21-L container was covered with a polyurethane plate with apertures for the plant to grow, for air to be supplied, for air samples to be taken and for the nutrient solution taken up by the plants to be replenished. The aperture for nutrient replenishment consisted of a hopper with an outlet arranged below the nutrient solution level in the container. An overflow aperture at the level of 15 L ensured that the intake for air sampling remained above the nutrient solution level. The overflow was also measured whenever the nutrient solution was replenished. Ambient air was pumped into the containers and blown into the nutrient solution at ground level, aerating and blowing out the CO_2_ of the nutrient solution. The air flow rate was controlled separately for each container by variable area flow meters (Westphal, Ottobrunn, Germany). The flow rate was adjusted to 100 L h^–1^, which ensured that no additional ambient air was able to enter the container, even when container air was sampled. In addition, this flow rate made sure that the CO_2_ concentration remained within the metering range of the sensor whilst maximizing the resolution of the measuring signal. A pump alternately sucked ambient air and air from the containers, controlled by magnetic valves (Sirai, Bussero, Italy). The air was pumped at a flow rate of 40 L h^–1^ into the CO_2_ sensor. The measurement signal stabilized after 2 min, resulting in a measurement cycle of 96 min for the 24 containers. The magnetic valves were controlled and the data recorded by a programmable data logger (TopMessage; Delphin Prozesstechnik, Bergisch Gladbach, Germany). Finally, CO_2_ gas exchange rates in the root zone were determined by calculating the difference between the CO_2_ concentrations in the air in the containers and the ambient air supplied, and the air exchange rate of the containers.

### Simultaneous measurement of CO_2_ gas exchange in the shoot and root zone using a cuvette

The CO_2_ gas exchange in a whole plant was measured in a virtually airtight cuvette (length 0·5 m, width 1·0 m, height 1·7 m) made from polymethyl acrylate, placed in a temperature-controlled room. The front side could be opened, enabling the plant to be set in the cuvette. A ventilator (1800 m^3^ h^–1^) first transported air from the cuvette for cooling through a heat exchanger, which was connected to a thermostat (T4600; Lauda Dr R. Wobser, Lauda-Königshofen, Germany). The air was then transported to an electric heater and subsequently redirected to the cuvette. This procedure enabled the temperature and air humidity to be controlled. Fresh air was added to the cuvette using a compressor, resulting in an overpressure of 200 Pa and an air exchange rate of 0·5 m^3^ h^–1^, which compensated for any leakage. Nine high-pressure sodium discharge lamps (SON-T Plus 600 W; Philips, Amsterdam, The Netherlands) were installed above the cuvette. In order to attain the target CO_2_ concentration in the cuvette, commercially pure CO_2_ was added to the cuvette during the light phase, compensating for the plants' uptake and leakage using a mass flow controller (5850E; Brooks Instrument, Veenendaal, The Netherlands). The CO_2_ concentration in the fresh air and cuvette air was measured using an infrared CO_2_ gas analyser (URAS 14; ABB Automation Products, Frankfurt am Main, Germany). The CO_2_ concentration was not controlled during the dark. The plants were grown in an aerated nutrient solution in a 13-L container made from stainless steel. The container was equipped for measuring CO_2_ gas exchange in the root zone, resembling the system in the greenhouse experiments. In this case, air from the cuvette was pumped into the root container and the continuously sampled air for measuring the CO_2_ concentration in the container was redirected to the container. The actual plant was suspended from a frame mounted on the container. The container stood on an electronic balance (IC64; Sartorius, Göttingen, Germany), enabling continuous measurement of the mass loss of the system, which may be attributable almost completely to plant transpiration. All measurements were recorded and control algorithms were run on a programmable data logger (TopMessage). The CO_2_ gas exchange in the root environment was calculated analogously to the greenhouse experiments; the CO_2_ gas exchange in the shoot environment was estimated from the CO_2_ supply rate, the ambient CO_2_ concentration and the air leakage rate, and the CO_2_ concentration and air flow rate originating from the root environment.

### Plant characteristics

Fruits were harvested when they had reached a mass of ∼400 g or when required for the purposes of the treatment. At the end of the greenhouse experiments, and once CO_2_ gas exchange had been measured in the CO_2_ gas-exchange cuvette, the length of all leaves was measured. This enabled the leaf area to be estimated using a formula predetermined in a laboratory using direct leaf area measurements (Schwarz and Kläring, 2001). The first leaves from the two side shoots in the greenhouse experiments or, in the case of cuvette plants, one leaf from a side shoot, and the second, fifth and eighth leaves from the main stem (counted from the top) were sampled and frozen at –20 °C in order to measure soluble solids concentration (SSC) at a later date. The plants were then completely harvested and divided into their organs, namely leaves, stems, fruit and roots. Complete roots and samples of leaves, stems and fruits were dried in a ventilated oven at 80 °C (fruit at 105 °C) for 2 d to estimate the dry matter content and dry matter of the organs. After defrosting, the leaves were divided into two (greenhouse) and three (cuvette) subsamples, and the sap was extracted by pressing the material in a cylinder using a force of ∼2 bar. SSC was measured for three replicates per subsample using a refractometer (PR-101α; ATAGO, Tokyo, Japan) and averaged over all subsamples. A carbon content of 40 % in the dry matter was assumed when the dry matter data were compared with the CO_2_ gas exchange measurements.

### Treatments in the long-term root-zone respiration experiments

Twenty-four cucumber plants were set in polyethylene containers on 6 February in experiment RZR1 and on 18 March 2008 in experiment RZR2. Whilst the young plants in experiment RZR1 were immediately set in the system for measuring root-zone gas exchange, in experiment RZR2 they were transferred to this system on 10 April. The experimental containers were arranged in eight blocks, each containing three containers. Additional plants were set as a border around the experiments in order to avoid edge effects. Once the fruit had set on the main stem (26 March in experiment RZR1, 30 April in experiment RZR2), three treatments were arranged in each block, focusing on a variation of the sinks for carbohydrates on the plants: (i) apart from the standard cultivation measures described above, no further plant organs were removed; (ii) in addition to the standard cultivation measures, all fruits on the main stem were removed and their mass was recorded; and (iii) in addition to the measures in treatment (ii), side shoots were cut off after the appearance of two leaves, and fruits were removed from the side shoots as soon as they started growing. The experiments were terminated 13 d after the treatments had commenced and plant characteristics were measured as described above. The data underwent analysis of variance. The differences between treatments were evaluated using Fisher's *F* procedure followed by Tukey's *T* procedure at a significance level of α = 0·05.

### Treatments in CO_2_ gas-exchange cuvette experiments

Seeds were sown on 21 June, 9 July, 27 July and 28 August 2007. The plants were grown in a greenhouse and then transferred to the gas-exchange cuvette. The four experiments started shortly after fruit growth began on 30 July, 27 August, 10 September and 24 October, respectively. Plants were transferred to the cuvette consecutively during the experiments. Thus, the fruit load of investigated plants usually increased over the course of the experiments. In addition, fruit load was varied by removing fruits at different stages. In experiment C1 (12 plants), fruits were removed from the plants at the harvest stage, which meant that only two of the 12 plants had no fruit (all fruits had reached the harvest stage) when they were transferred to the cuvette. In experiment C2, alternately either none or all of the fruits were removed just before transferring the plants to the cuvette (a total of six plants). In experiment C3 (nine plants), the fruits were left on the plants or removed 0 or 4 d prior to transferring the plants to the cuvette. The same procedure was conducted in experiment C4 (four plants), in which the last plant had fruit again. Thus, a total of 13 plants had no fruits when they were transferred to the cuvette; 18 plants had fruits, six of them having a small fruit load of <20 g dry matter. The environmental conditions in the gas-exchange cuvette were set at 25 °C and 70 % relative humidity both day and night. During the 12 h-light phase, the plants were illuminated using four lamps, resulting in a photosynthetic photon flux density in the middle of the (empty) cuvette of 600 μmol m^–2^ s^–1^. The CO_2_ concentration during the light phase was controlled at 400 μmol mol^–1^; during the dark phase it levelled out at ∼600 μmol mol^–1^ due to the lack of CO_2_ absorber. The plants were placed in the cuvette 1–2 h after the light phase started. They were removed from the cuvette by the end of the dark phase of the second or third day, and analysed as described above. The first light and dark phases were considered to be adaptation stages; only the second light and dark phases were used to evaluate the data by regression analysis.

## RESULTS

### Long-term root-zone respiration

**Fig. 1.**
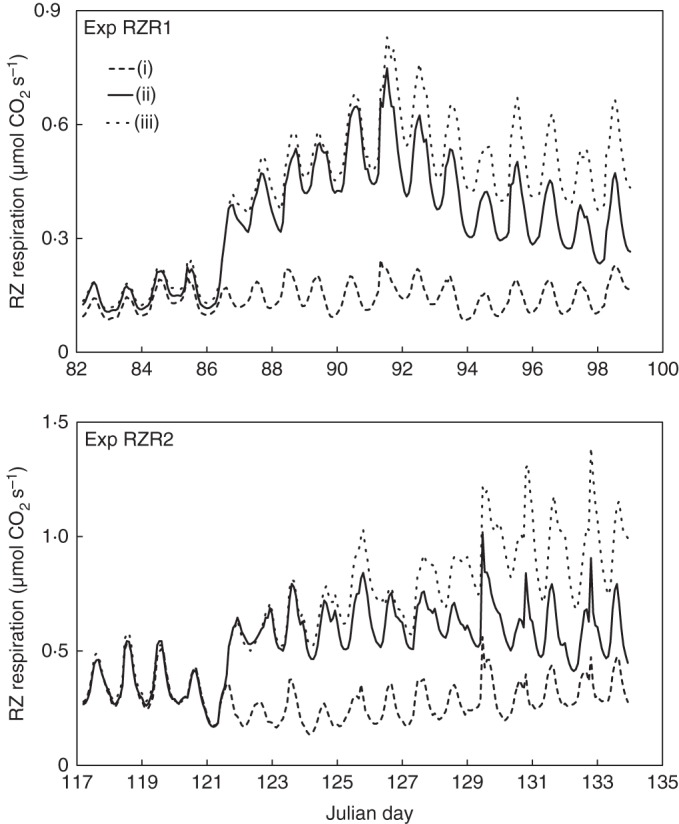
Root-zone (RZ) respiration of cucumber plants affected by different constraints on carbohydrate sinks. Treatments [(i) no fruit removed, (ii) fruit removed and (iii) fruit removed and side shoots cut] started on the morning of day 86 in experiment RZR1 and day 121 in experiment RZR2. The numbers on the axis mark the start of the day. Data are mean values of root-zone respiration per plant. The mean relative standard error (n = 8) of all measurements was <7 % in experiment RZR1 and 5, 9 and 13 % in treatments (i), (ii) and (iii), respectively, in experiment RZR2.

The same pattern of CO_2_ gas exchange in the root environment was observed in both greenhouse experiments. The daily course of gas exchange followed the course of the temperature in the containers (temperature data not shown). When fruits were removed from the plants, gas exchange rose suddenly and dramatically by a factor of 2, and later even by a factor of 3 compared with plants that retained the sinks (Fig. 1, Table 1). Approximately 1 week after starting the treatments, the gas exchange of treatment (ii) – side shoots with fruits that were allowed to grow – decreased significantly compared with treatment (iii) – side shoots terminated, zero fruit growth. However, it remained well above the level of the control treatment (i), in which nothing was removed over the experimental period.

**Table 1.** Root-zone CO_2_ release and water consumption cumulated over the treatment period and growth characteristics measured at the end of the treatment period in experiment RZR1 (first line) and experiment RZR2 (second line) of the treatments: (i) no fruit removed, (ii) fruit removed and (iii) fruit removed and side shoots cut. Fruit dry matter is the sum of fruit dry matter removed at the start of treatments (ii) and (iii), harvested during treatment (i) and remaining on the plants at the end of the experiment. The dry matter content of plant compartments is given as g dry matter per g fresh matter. The dry matter equivalent of root-zone gas exchange was calculated assuming a carbon content of 40 % in the dry matter

	Treatment		
Characteristic	(i)	(ii)	(iii)
Root-zone CO_2_ release per plant, g	7·3^a^	20·3^b^	24·9^c^
	13·4^a^	29·4^b^	38·7^c^
Water consumption per plant, kg	14·4^a^	13·5^a^	12·9^a^
	33·8^b^	33·1^b^	27·2^a^
Leaf dry matter per plant, g	47·5^a^	79·0^b^	80·3^b^
	64·7^a^	108·8^b^	98·9^b^
Leaf dry matter content, g g^–1^	0·079^a^	0·090^b^	0·105^c^
	0·088^a^	0·094^a^	0·109^b^
Leaf soluble solid concentration, °Brix	3·08^a^	3·44^a^	4·48^b^
	3·89^a^	4·05^a^	4·95^b^
Leaf area per plant, m^2^	2·53^a^	3·21^b^	2·75^ab^
	2·30^a^	2·90^b^	2·25^a^
Specific leaf area (fresh), dm^2^ g^–1^	0·420^b^	0·364^a^	0·362^a^
	0·314^b^	0·256^a^	0·250^a^
Specific leaf area (dry), m^2^ g^–1^	0·054^c^	0·041^b^	0·035^a^
	0·036^c^	0·027^b^	0·023^a^
Stem dry matter (main + side shoots) per plant, g	17·9^a^	43·8^c^	27·8^b^
	23·9^a^	55·5^c^	35·1^b^
Stem dry matter content, g g^–1^	0·063^a^	0·069^b^	0·084^c^
	0·069^a^	0·072^a^	0·091^b^
Root dry matter per plant, g	6·7^a^	13·6^b^	17·8^c^
	9·7^a^	18·2^b^	23·5^c^
Root dry matter content, g g^–1^	0·031^a^	0·031^a^	0·032^a^
	0·033^b^	0·030^a^	0·029^a^
Fruit dry matter per plant, g	129·8^b^	64·9^a^	62·8^a^
	148·6^b^	42·3^a^	34·2^a^
Total dry matter per plant, g	202·0^a^	201·3^a^	188·7^a^
	246·9^b^	224·7^b^	191·7^a^
Dry matter + dry matter equivalent of root-zone gas exchange per plant, g	206·9^a^	215·1^a^	205·6^a^
	256·0^b^	244·8^b^	218·1^a^

Numbers followed by the same letter do not differ significantly (Tukey's multiple range procedure at a significance level of α = 0·05 and *n* = 8 replications).

**Fig. 2.**
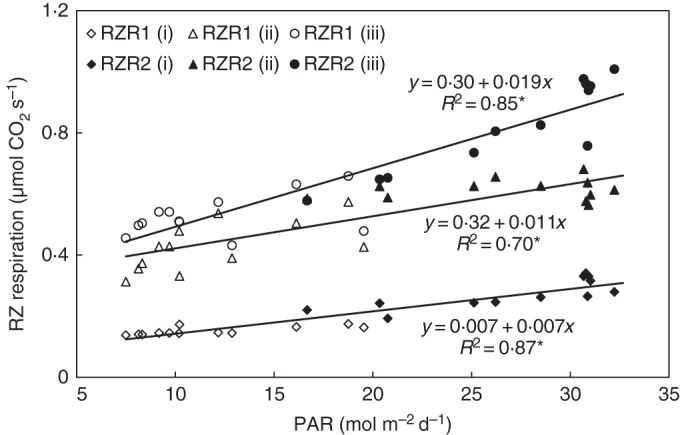
Effect of daily PAR on the 24-h mean of root-zone (RZ) respiration per plant in experiments RZR1 and RZR2. Coefficients of determination (*R*^2^) marked by an asterisk differ from zero at a significance level of α = 0·05.

While the root-zone gas exchange of treatment (iii) increased in the first half of experiment RZR1 and decreased in the second half, it was lower in the first half than in the second half of experiment RZR2. This can be explained by the average PAR, which was 14·9 and 8·8 mol m^−2^ d^−1^ in the first and second halves of experiment RZR1 and 23·3 and 30·7 m^−2^ d^−1^ in the first and second halves of experiment RZR2, respectively. Figure 2 emphasizes this effect, explaining 87, 70 and 85 % of the variance in root-zone respiration by the variation in mean daily PAR in treatments (i), (ii) and (iii), respectively. Eliminating the first 2 d after the start of the treatments (for possible adaptation), the coefficient of determination increased to 0·92 in treatment (iii) and 0·77 in treatment (ii).

Removing the fruits also resulted in a significant increase in the vegetative growth of the plants (Table 1). In both treatments (ii) and (iii), leaf dry matter was 53–69 % higher compared with the control treatment (i). Surprisingly, no difference was observed in leaf dry matter between treatments (ii) and (iii), although allowing the side shoots to grow in treatment (ii) led to an ∼25 % larger leaf area than that in treatment (iii). In the control treatment (i), almost all of the carbohydrates were distributed to the fruit, meaning that side shoot growth was limited and leaf area did not differ from treatment (iii), in which the side shoots had been cut. In both treatments (ii) and (iii), specific leaf area (SLA) in relation to fresh matter decreased by the same proportion compared with the control treatment (i); that is, the leaves became thicker. In relation to dry matter, however, a further decrease in SLA was observed from treatment (ii) to treatment (iii) due to the marked increase in leaf dry matter content in treatment (iii). This high density of carbohydrates in the leaves was also reflected by a significant increase in SSC in the leaf in treatment (iii) compared with treatment (i) (Table 1).

A similar pattern was observed for the stems. Stem dry matter content increased dramatically if there were fewer sinks on the plant (Table 1). However, there was less stem dry matter in treatment (iii) than in treatment (ii) because the higher dry matter content in treatment (iii) did not fully compensate for the unrestricted side shoot growth in treatment (ii).

Restricting shoot growth led to a dramatic stimulation of root growth (Table 1). As a result, root dry mass doubled in treatment (ii) compared with the control treatment (i) in a matter of 13 d, and increased by a factor of 2·5 in treatment (iii). The root dry matter content was virtually unaffected, apart from a slight decrease in treatments (ii) and (iii) compared with the control treatment (i) in experiment RZR2, probably due to the lower dry matter content of newly grown young roots. The leaf/root ratio based on dry matter in treatment (iii) was 64 % of that in the control treatment (i); calculated on the basis of leaf area, it fell to as little as 41 % (data not shown, derived from Table 1).

Overall, vegetative plant dry matter was ∼87 % higher in treatment (ii) compared with the control treatment (i). In treatment (iii) it increased by 75 % in experiment RZR1 and by 60 % in experiment RZR2 (data not shown, derived from Table 1).

In line with the objectives of the experiments, fruit growth was significantly restricted in treatments (ii) and (iii). While after the start of the treatments in (iii) no fruit growth was seen, in treatment (ii) fruit dry matter on the side shoots averaged 3·6 and 7·2 g per plant in experiments RZR1 and RZR2, respectively. In terms of total dry matter, however, vegetative growth almost fully compensated for the lack of fruit growth and side shoot growth in experiment RZR1, whilst in experiment RZR2 there was only partial compensation for the restriction of fruit and side shoot growth (Table 1). This figure remained unchanged when the dry matter equivalent of the root-zone gas exchange was added (Table 1).

The treatments appeared to have no impact on water consumption in experiment RZR1. In experiment RZR2, however, water uptake in treatment (iii) was 20 % less than that in the control treatment (i) (Table 1).

The microclimate in the greenhouse differed in the two experiments due to the staggered planting dates. The average PAR at the level of the tops of the plants and the mean temperature over the growing period were 9·3 mol m^–2^ d^–1^ and 20·7 °C in experiment RZR1 and 17·5 mol m^–2^ d^–1^ and 21·2 °C in experiment RZR2. Consequently, dry masses of all organs, leaf and stem dry matter contents, leaf SSC and, in particular, water consumption were higher in experiment RZR2 than in experiment RZR1, whereas SLA was lower.

### Simultaneous measurement of CO_2_ gas exchange in the shoot and root zone

**Fig. 3.**
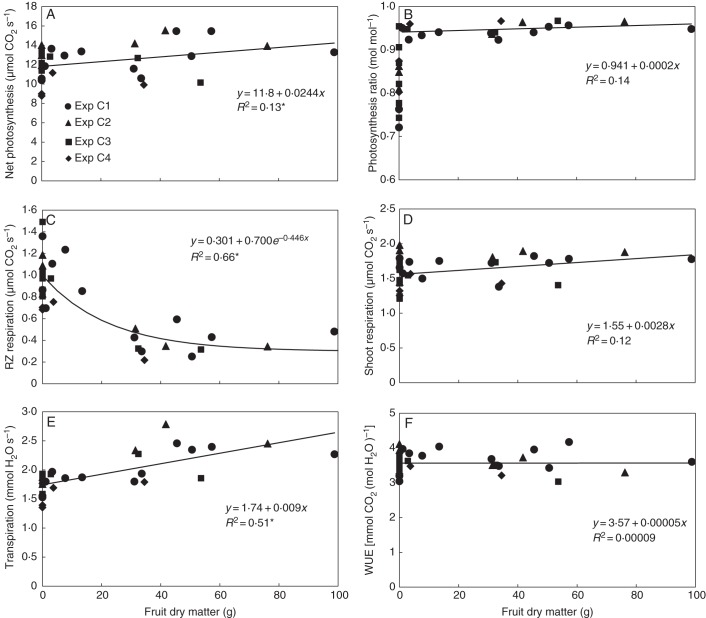
Effect of fruit load on mean net photosynthesis of the shoot during the light phase (A), the evening/morning ratio of net photosynthesis, where the correlation includes only the data points of fruit-bearing plants (B), mean 24-h root-zone (RZ) respiration (C), mean shoot respiration during the dark phase (D), mean transpiration during the light phase (E) and water use efficiency (WUE), which includes the daily water and CO_2_ balance of the shoot and root (F). All data relates to one plant. Coefficients of determination (*R*^2^) marked by an asterisk differ from zero at a significance level of α = 0·05.

The sink–source status of the plants was modified by partly removing the fruit before taking measurements. Nevertheless, net photosynthesis was only affected slightly by the total fruit load of the plants (Fig. 3A). The effect of fruit load on photosynthesis by the end of the light phase was more pronounced. The absence of fruit resulted in an average 12 % reduction in the evening/morning ratio of net photosynthesis, which ranged from 72 to 95 %. In fruit-bearing plants, however, this ratio was within a much narrower range (92·3–96·7 %) regardless of the plant's fruit load (Fig. 3B).

**Fig. 4.**
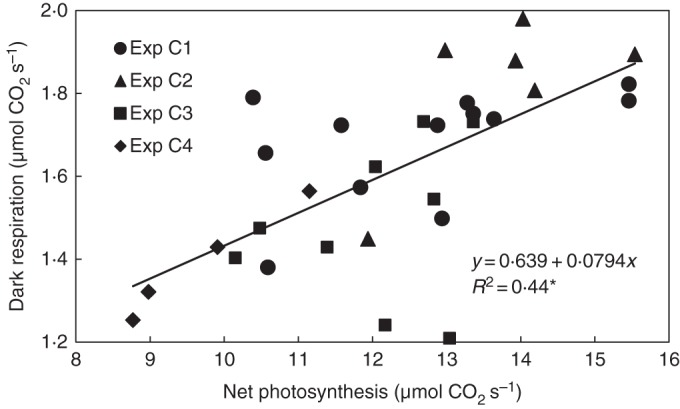
Effect of mean net photosynthesis of the shoot during the day on mean dark respiration of the shoot during the following night. Data relate to one plant. The coefficient of determination (*R*^2^) differs from zero at a significance level of α = 0·05.

Root-zone respiration was around 0·4 μmol s^–1^ when the fruit load exceeded 20 g dry matter. Unlike photosynthesis, root-zone respiration increased significantly when the fruit load was small (Fig. 3C). In contrast to root-zone respiration, no clear impact of fruit load on shoot respiration was observed (Fig. 3D). Mean shoot respiration in the dark phase was correlated with the photosynthesis in the preceding light phase (Fig. 4).

The removal of fruit reduced transpiration significantly (Fig. 3E). In the daily balance, however, water use efficiency (WUE) was unaffected, due to the decrease in the daily carbon balance by the high root-zone respiration with zero and low fruit load (Fig. 3F).

**Fig. 5.**
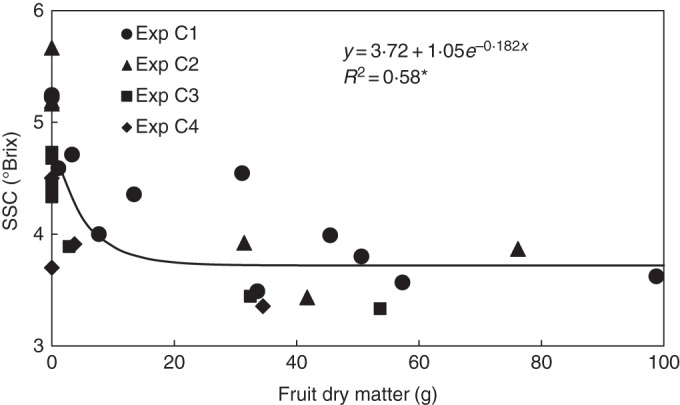
Effect of fruit load on the soluble solid concentration (SSC) in the leaf. The coefficient of determination (*R*^2^) differs from zero at a significance level of α = 0·05.

**Fig. 6.**
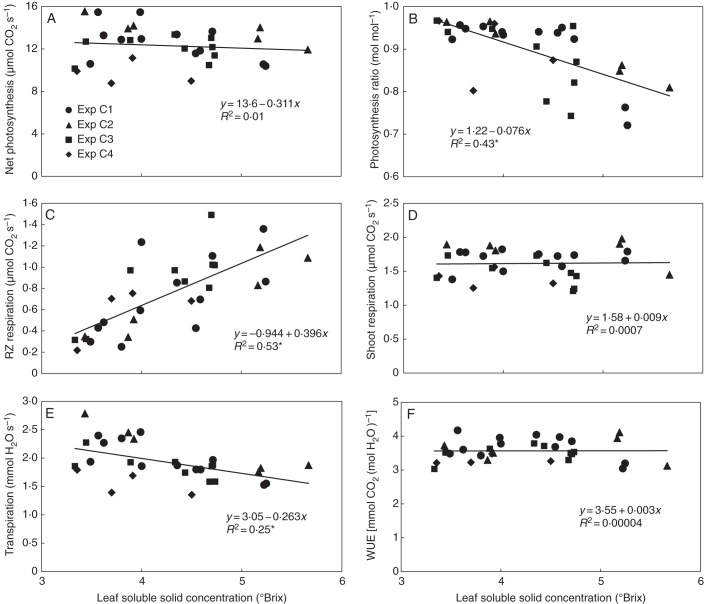
Effect of leaf carbohydrate status, expressed as soluble solid concentration in the leaf, on mean net photosynthesis of the shoot during the light phase (A), the evening/morning ratio of net photosynthesis (B), mean 24-h root-zone (RZ) respiration (C), mean shoot respiration during the dark phase (D), mean transpiration during the light phase (E) and WUE, which includes daily water and CO_2_ balance of the shoot and root (F). All data relates to one plant. Coefficients of determination (*R*^2^) marked by an asterisk differ from zero at a significance level of α = 0·05.

**Fig. 7.**
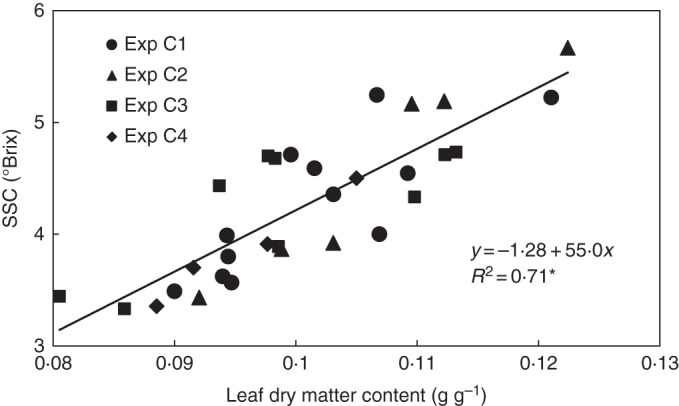
Correlation between leaf dry matter content and soluble solid concentration (SSC) in the leaf. The coefficient of determination (*R*^2^) differs from zero at a significance level of α = 0·05.

The SSC in the leaf reflected the results for root-zone respiration: it was almost constant at 3·7 °Brix for a fruit load exceeding 20 g dry matter per plant, but increased significantly when the fruit load fell below 20 g (Fig. 5). Plotting the CO_2_ and H_2_O gas exchange data against SSC therefore produced patterns similar to those in Fig. 3, with linearization of the relationships for the evening/morning ratio of photosynthesis and for root-zone respiration (Fig. 6). Although different leaves of the plant were measured, SSC strongly correlated with leaf dry matter content (Fig. 7). For this reason, replacing SSC in Fig. 6 with leaf dry matter content made no difference to the patterns (data not shown).

In Figs 3, 4 and 6, the gas-exchange data relate to one plant. Relating photosynthesis and transpiration data to leaf area and respiration data to the dry masses of the corresponding organs did not change the overall figure (data not shown).

The gas-exchange data were also affected by the intensity of solar radiation received by the plants during cultivation in the greenhouse prior to measurements being taken in the gas-exchange cuvette. Plants grown from the last planting date in particular (experiment C4) therefore exhibited lower gas-exchange activity (Figs 3 and 6).

## DISCUSSION

### Root-zone respiration

The most interesting and most definite result of constraining sink strength in cucumber plants by removing fruit and restricting shoot growth was the sudden doubling of CO_2_ gas exchange in the root zone in both greenhouse experiments. Similar results were obtained when plants were cultivated in an organic substrate (H.-P. Kläring, unpubl. res.). Over the next few days, the root-zone gas exchange rose by a factor of 3 compared with the full sink strength control treatment (Fig. 1). These time courses were modified by a significant effect of irradiance on root-zone respiration (Fig. 2). On average, the same scale of increase was observed in the CO_2_ gas-exchange cuvette, where some, none or all fruit had been removed immediately or several days before taking measurements. Plants with a low fruit load (<20 g dry matter) also exhibited enhanced root-zone gas exchange. A review of the literature revealed no evidence of reports on the impact of sink limitation on root (zone) respiration.

In the search for explanations of the observed phenomenon, the key components of CO_2_ release in the root zone need to be evaluated. In the case of non-limited sinks, root-zone CO_2_ release per plant averaged 0·39 μmol s^–1^ in the cuvette experiments (plants with fruit dry matter >20 g); it was 0·15 and 0·27 μmol s^–1^ in the greenhouse experiments (Figs 1 and 3C). These figures correspond to root-zone respiration per plant of 1·47 g CO_2_ d^–1^ in the cuvette and 0·57 and 1·03 g d^–1^ in the greenhouse experiments. In most models, total root respiration is described as the sum of two important components: maintenance and growth respiration (McCree, 1970; Amthor, 2000). The maintenance respiration rate has been reported to be 0·015 g C (g C)^–1^ d^–1^ at 25 °C (Marcelis, 1994). In relation to an average root mass of 14·3 g for fruit-bearing plants in the cuvette experiments (data not shown), maintenance respiration accounts for a daily CO_2_ root gas exchange of 0·31 g. An approximate estimate of growth respiration can be obtained from the mean daily carbon balance of the fruit-bearing plants in the cuvette experiments: the mean daily carbon net assimilation derived from data in Fig. 3 is 5·45 g. Assuming a fraction of 3·5 % is attributable to root growth [ratio of root to total dry matter in the greenhouse experiments (Table 1)] and a dry matter requirement for root growth of 1·3 g g^–1^ (Cannell and Thornley, 2000), a daily fraction of 0·057 g carbon or 0·21 g respired CO_2_ is yielded. Specifically in relation to roots, a further fraction of carbon use must be considered, namely the energy requirement for active ion uptake (Van der Werf *et al*., 1988). Measuring a mean daily dry matter net production of 13·6 g (derived from Fig. 3) and a nitrogen concentration in dry matter of ∼3·5 %, and assuming respiration costs of 1·25 g CO_2_ per g NO_3_ nitrogen taken up (Cannell and Thornley, 2000) yields 0·60 g respired CO_2_. To simplify matters, NH_4_ uptake is neglected due to the very low NH_4_ supply in the nutrient solution (De Kreij *et al*., 1997). Respiration costs for the uptake of other ions are also disregarded because of their relatively low impact (Cannell and Thornley, 2000). The respiration costs for nitrate reduction (Marschner, 1995) are also low, and can be ignored when nitrate reduction for root biomass growth only is included.

Summarizing, on average, 21 % of the measured root-zone respiration of plants with non-limited sinks in the cuvette experiments can be attributed to maintenance, 14 % to root growth and 41 % to nutrient uptake.

Therefore, only 24 % remains for other contributory factors. Potentially significant additional components could be the alternative respiratory pathway, which, however, gains in importance in the case of limited sinks for carbohydrates (Lambers, 1982), and the exudation of organic components by the roots and their metabolism by micro-organisms in the root environment. Other CO_2_ sources, such as the microbial respiration of dead plant residues, can be neglected due to their low turnover rates (Kuzyakov, 2006).

Similar percentages of CO_2_ release components can be assumed for the greenhouse experiments. The lower total root-zone respiration in greenhouse experiment RZR1 compared with experiment RZR2 can be attributed to the season. Here, the lower levels of irradiance resulted in a lower rate of photosynthesis and smaller roots, which meant that less energy was consumed for maintenance, growth and nutrient uptake.

In the case of fruit removal, sinks of the shoot were limited and root-zone respiration increased immensely. There are a number of hypotheses that could explain this phenomenon: (1) root maintenance respiration rate increased; (2) the alternative respiratory pathway was stimulated; (3) root growth was stimulated; (4) active nutrient uptake and nitrate reduction were enhanced; (5) root exudation and microbial activity in the root zone increased. These hypotheses are discussed below.

*Hypothesis (1)*. Maintenance respiration increases exponentially with increasing temperature (Amthor, 1989). In the greenhouse experiments, root-zone respiration followed the daily course of temperature (Fig. 1). Since dark respiration of the shoot increased only slightly as the fruit load increased, which can probably be attributed to respiration of the actual fruit (Marcelis and Baan Hofman-Eijer, 1995), an increase in root maintenance respiration was unlikely to be responsible for the enhanced root-zone CO_2_ gas exchange immediately after fruit removal (Fig. 1). In contrast, Den Hertog *et al*. (1993) found increased dark respiration of the shoot but observed no change in root respiration in *Plantago major* when the carbon source was increased by raising the CO_2_ concentration. On the other hand, increasing photosynthesis by irrigating part of the root system of ponderosa pines in a semi-arid forest resulted in a significant increase in the respiration of the rhizosphere (Irvine *et al*., 2005). As in the present study, however, the measurements included both root respiration and the decomposition of root exudates by micro-organisms in the rhizosphere. In the medium term, fruit removal greatly stimulated root growth (Table 1) and thus maintenance respiration increased with root size. The order of magnitude, however, was far below the increased root-zone respiration. For example, the total root-zone respiration in treatment (iii) by the end of experiment RZR2 was 3·62 g CO_2_ d^–1^, whereas the estimated maintenance respiration based on a root size of 23·5 g (Table 1) was 0·52 g d^–1^, which is only a small fraction (14 %) of the total root-zone respiration.

*Hypothesis (2)*. Increasing respiratory rates may lead to an over-reduction of components of the electron transport chains (Turrens, 2003), resulting in the synthesis of reactive oxygen species (Moller, 2001). The non-phosphorylating alternative oxidase pathway avoids this over-reduction, and may therefore act as an overflow for energy that cannot be used for biosyntheses and growth (Lambers, 1982). Modulation of the different respiratory pathways has mainly been reported for source organs and increasing sources (González-Meler *et al*., 2001; Millenaar and Lambers, 2003). Gandin *et al*. (2009) also reported a marked stimulation of the alternative respiratory pathway, and thus an increase in total respiration rate, for a sink organ, the bulb of *Erythronium americanum*, when source activity was increased by raising the CO_2_ concentration. In parallel, however, the alternative pathway activity of the leaves increased (Gandin *et al*., 2009), rendering hypothesis (2) unsuitable for explaining the increased root-zone gas exchange in combination with unaffected shoot dark respiration. In any case, hypothesis (2) is also inadequate for accounting for the scale of increase in the root-zone CO_2_ gas exchange, because alternative pathway respiration is no more than 30 % of total respiration (Gandin *et al*., 2009).

*Hypothesis (3)*. Stimulating root growth by removing sinks on the shoot would increase growth respiration in the roots. Thus, in greenhouse experiment RZR2, root growth rate in treatment (iii) with strongly restricted sinks was on average 1 g d^–1^ greater than in treatment (i), in which sinks of the shoot were not restricted. This may account for 0·44 g CO_2_ greater growth respiration in (iii) compared with (i), which is ∼20 % of the difference in total root-zone respiration at the end of the experiment. The effect was probably more pronounced towards the end of the experiment because immediately after removing the sinks latent buds require a certain amount of time before their growth is stimulated.

*Hypothesis (4)*. In contrast to hypotheses (1) to (3), there is no indication that respiration related to nutrient uptake may have increased when the sinks of the shoot were constrained. Dry matter production either remained unaffected or decreased (experiment RZR2).

*Hypothesis (5)*. Since the quantitative effects of root respiration types (1) to (4) are an inadequate explanation of all the differences in root-zone respiration between sink-limited and sink-unlimited plants, the exudation of organic compounds by the roots and their decomposition by micro-organisms in the root zone remains the only possible activity that can account for almost all the increase in the measured CO_2_ gas exchange immediately after sink removal, and also for the largest fraction later on.

Similar results were obtained when estimating the contributions made by the different compounds of root-zone respiration in the sink-limited plants on the second day in the cuvette experiments based on data from Fig. 3. A dramatic increase in root growth and the ‘remaining’ fraction was observed in both absolute terms and percentages, compared with the sink-unlimited plants (Table 2). While the ‘remaining’ fraction in the sink-unlimited plants constitutes only 1 % of CO_2_ fixed in daily gross photosynthesis, estimated as daytime net photosynthesis plus dark respiration (data from Figs 3A, D), this ratio yielded a considerable value of 8 % in the sink-limited plants.

**Table 2.** Estimated fractions of different components of root-zone gas exchange measured in the gas-exchange cuvette in sink-unlimited plants (fruit dry matter >20 g) and sink-limited plants (fruit completely removed). Data are given in g CO_2_ d^–1^ per plant (%)

Compound	Sink-unlimited	Sink-limited
Total	1·47 (100)	3·93 (100)
Maintenance	0·31 (21)	0·39 (10)
Growth	0·21 (14)	1·00 (25)
Nutrient uptake	0·60 (41)	0·46 (12)
Remaining	0·35 (24)	2·07 (53)

Adding 4 g of glucose and 4 g of fructose to the nutrient solution in a further experiment, which corresponds to ∼12 g of CO_2_ when metabolized by the microorganisms, resulted in increased root-zone respiration in fruit-bearing plants by a factor of 2 within a few hours and a factor of 6 within 1 d. After ∼4 d, this raised level of root-zone respiration had disappeared (unpublished data). This verifies the presence of micro-organisms and their ability to metabolize a suddenly greater amount of organic compounds rapidly (Jones *et al*., 2004; Boddy *et al*., 2006). Thus, root exudation appeared to be the first response of plants to a sudden sink reduction. It can be considered as a carbon overflow for tackling the sudden imbalance of carbon sinks and sources. This process is likely to be much faster than the other responses, such as stimulating root growth, increasing the dry matter content of leaves and stems or changing root respiration (Hill *et al*., 2007).

Root exudation has been reported to have varying importance in the carbon balance of plants, ranging from just a few percent to 40 % of total assimilated carbon (Grayston *et al*., 1996). The higher values were yielded in perennial plants, the lower values under sterile conditions. Jones *et al*. (2004) distinguished between two classes of root exudation, namely leakage of compounds as a result of passive diffusion over which the plant exerts little control, and exudation of specific compounds with a specific function over which the plant exerts control. Root exudation acting as an overflow of carbohydrates, as hypothesized in this study, is assumed to occur by passive diffusion (Nguyen, 2003). There is large body of evidence that suggests that sugars dominate root exudates (Lugtenberg *et al*., 1999) under most conditions. Thus, strong limitations in sinks of the shoot for carbohydrates may result in their enhanced translocation to the roots, a higher concentration of soluble carbohydrates in the root cytoplasm and, finally, greater diffusion due to the increasing gradient between solute concentrations inside and outside the roots (Nobel, 1983; Jones *et al*., 2004). However, the nature of any benefits to plants from such exudation is still being debated (Nguyen, 2003). One advantage could be that the early end-product inhibition of the photosynthesis is avoided.

The exudation of specific compounds, usually associated with nutrient acquisition (e.g. Jones *et al*., 2004), plays an insignificant role in properly controlled, aerated nutrient solutions. This is supported by the very low total root-zone CO_2_ gas exchange in the sink-unlimited plants in all experiments (Figs 1 and 3C) and the low fraction of 1 % of gross photosynthesis, which may be attributed to the exudation of specific compounds and diffuse exudation coinciding with the range of 0·5–1·5 % reported by Farrar *et al*. (2003) for experiments performed in hydroponics. When all fruit was removed, however, root-zone respiration increased markedly and it is thought that 8 % of gross photosynthesis is lost by exudation according to the above estimates (Table 2). This amount is much higher than the figures usually reported in the literature for experiments in hydroponics. It lies within the range published for experiments carried out in soil (Jones *et al*., 2004). It must also be emphasized that the effect of the strong limitations of sinks on root-zone respiration has not been included in previously published reviews.

### Dry matter allocation

There is evidence of the dominance of attracting assimilates of fruit over vegetative organs in general (e.g. Wardlaw, 1990) and specifically in cucumber (Marcelis, 1992). Removing the fruit redirected carbohydrates to the vegetative compartments, stimulating their growth. Thus, any carbohydrates not required for fruit growth were used to increase leaf thickness and the dry matter content of stems and leaves (Table 1). Moreover, root growth increased dramatically even with limited leaf growth in treatment (iii) (Table 1). This resulted in a dramatic decrease in the leaf/root ratio, which contradicts the commonly accepted rules for leaf and root development of allometry (e.g. Pearsall, 1927; Poorter and Nagel, 2000) and functional equilibrium (e.g. Brouwer, 1963); i.e. from the perspective of nutrient and water uptake there was no need for enhanced root growth.

### Photosynthesis

In contrast to effects on root-zone respiration and dry matter distribution, the effect of limiting sinks on the photosynthetic characteristics of cucumber plants was very weak. This observation is in full agreement with Marcelis (1991), who found a reduction in leaf photosynthesis in adult cucumber plants only 2 weeks after fruit removal. A rough estimate of the contribution of the fruit to the total green surface of the plants (fruit + upper and lower leaf surfaces) resulted in a 1 % increase per 10 g of fruit dry matter. Thus, the marginal increase in photosynthesis with increasing fruit mass can be attributed in part to the photosynthesis of the green fruit itself (Marcelis and Baan Hofman-Eijer, 1995). It seems that in most conditions the leaves, stems and roots still provided sufficient sinks for carbohydrates to avoid early feedback inhibition of photosynthesis, particularly when there was still a small fruit load, and shortly after fruit removal.

Over the longer term, photosynthesis was sometimes inhibited, particularly by the end of the light period (Fig. 3B). The length of the delay of this inhibition depends on the state of the plant at fruit removal and the environmental conditions (particularly PAR). In greenhouse experiment RZR1, the total dry matter of the plants plus the root-zone respiration dry matter equivalent, and thus photosynthesis, were not affected by fruit removal. In contrast, in experiment RZR2, which involved significantly higher irradiance, this characteristic was reduced by 15 %, coinciding with results by Marcelis (1991).

Increased SSC also indicates changes in the leaves' osmotic potential, which may have led to the observed decrease in transpiration (Table 1, Figs 3E and 6E). Furthermore, the decrease in transpiration following fruit removal suggests changes in stomatal conductance since all environmental conditions were kept constant. Interestingly, water use efficiency of the complete carbon balance was unaffected by fruit removal (Figs 3F and 6F). Whether this happened by accident or by principle cannot be substantiated using the results of this study.

### Conclusions

The most interesting and concrete result of constraining sink strength in cucumber plants by removing fruit was the sudden doubling of CO_2_ gas exchange in the root zone, which is thought to be the result of the exudation of organic compounds by the roots and their decomposition by micro-organisms. This hypothesis requires further experimental evidence. Root exudation as carbon losses may have become a considerable factor in the carbon balance in the range below 20 g fruit dry matter per plant, i.e. below a plant fruit load of ∼700 g. It would therefore make sense to include carbon leakage by root exudation in cucumber production models. In contrast, the inhibition of photosynthesis was measurable only at zero fruit load, which does not occur in cucumber production systems, and models that estimate production can therefore ignore (end-product) inhibition of photosynthesis.
